# Design optimization and clinical prospects of a novel portable split-type negative pressure thoracic drainage device

**DOI:** 10.3389/fmed.2026.1778361

**Published:** 2026-05-14

**Authors:** Shaoqing Huang, Jie Li, Haibo Shen, Yang Yang

**Affiliations:** 1 Department of Thoracic Surgery, Ningbo No. 2 Hospital, Wenzhou Medical University, Ningbo, China; 2 Department of Thoracic Surgery, Shanghai Pulmonary Hospital, School of Medicine, Tongji University, Shanghai, China

**Keywords:** 3D printing, closed thoracic drainage, enhanced recovery after surgery (ERAS), portable device, split-type design

## Abstract

**Objective:**

To overcome the clinical limitations of traditional wall-mounted negative pressure drainage devices, which restrict early postoperative mobility and may delay enhanced recovery, this study aimed to optimize a self-developed portable thoracic drainage system through iterative design improvements.

**Methods:**

A novel second-generation portable negative pressure thoracic drainage device was developed based on a first-generation integrated design. The second-generation device employs a split-type architecture, wherein the negative pressure pump, power module, and optimized noise reduction system are integrated into an independent portable host via 3D printing technology. A flexible attachment method using double-sided adhesive tape and 3M hook-and-loop fasteners allows the host to be securely yet removably attached to the side of a standard three-chamber drainage bottle. The design prioritizes operational simplicity and clinical workflow compatibility.

**Results:**

The optimized device features a compact host measuring 139.6 × 95.6 × 35 mm and weighing about 400 g. The maximum negative pressure intensity main depends on the height of the water column in the three-chamber closed drainage bottle (close to 20 cm H₂O), host unit noise below 55 dB at a 3 L/min flow rate, and a battery life of 6–10 h. The estimated manufacturing cost is about 500 Chinese Yuan (~70 USD) per device unit, with a single-use consumable cost (drainage bottle) of about 45 Chinese Yuan (~6.3 USD).

**Conclusion:**

This study proposed the concept of a novel type of portable negative pressure thoracic drainage device and designed and manufactured a sample. In the next step, we will continue to improve this novel device and plan to apply it in clinical practice.

## 1 Introduction

### 1.1 Clinical background and practical need

**Figure 1:**
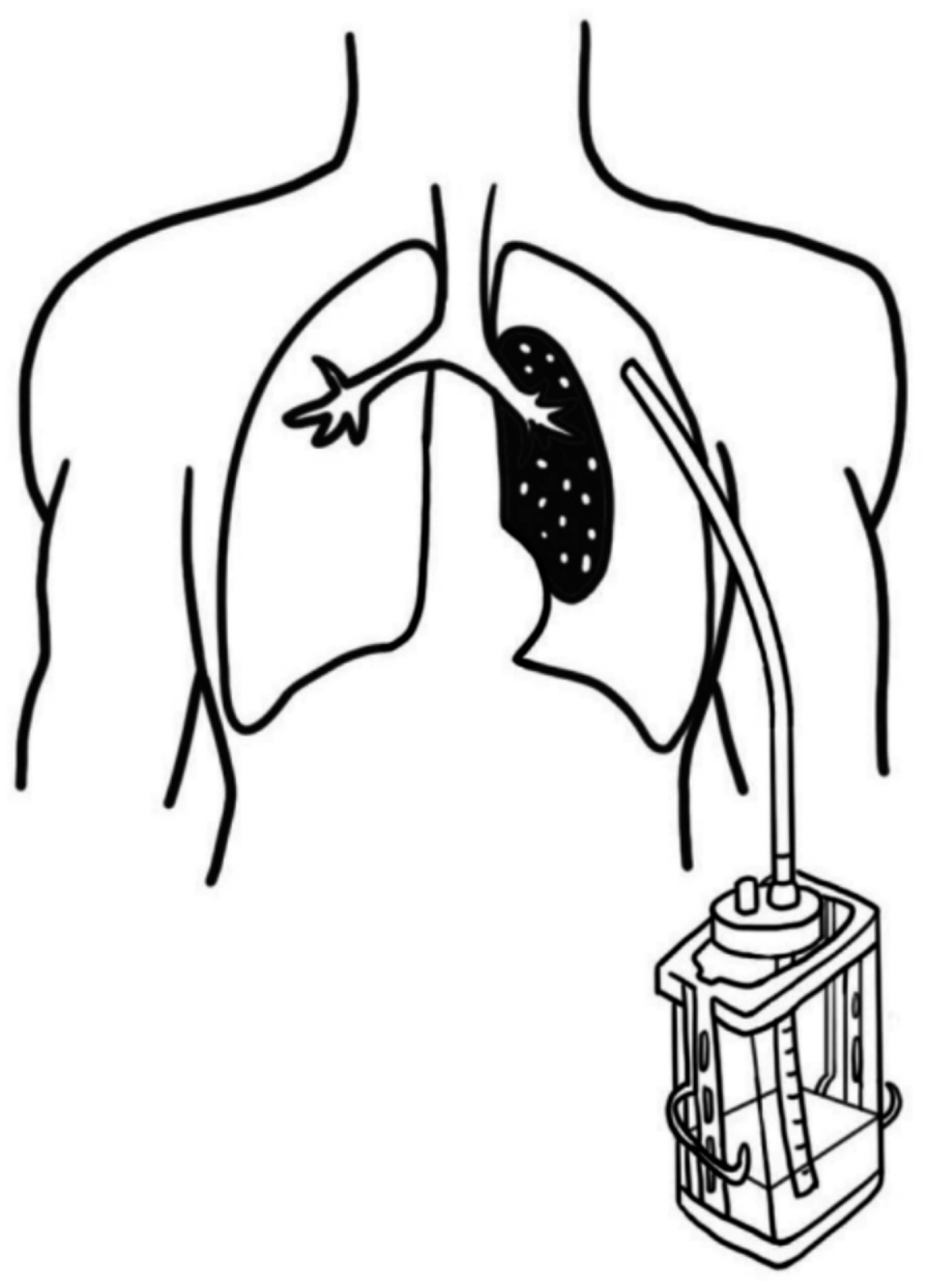
Chest closed drainage.

**Figure 2:**
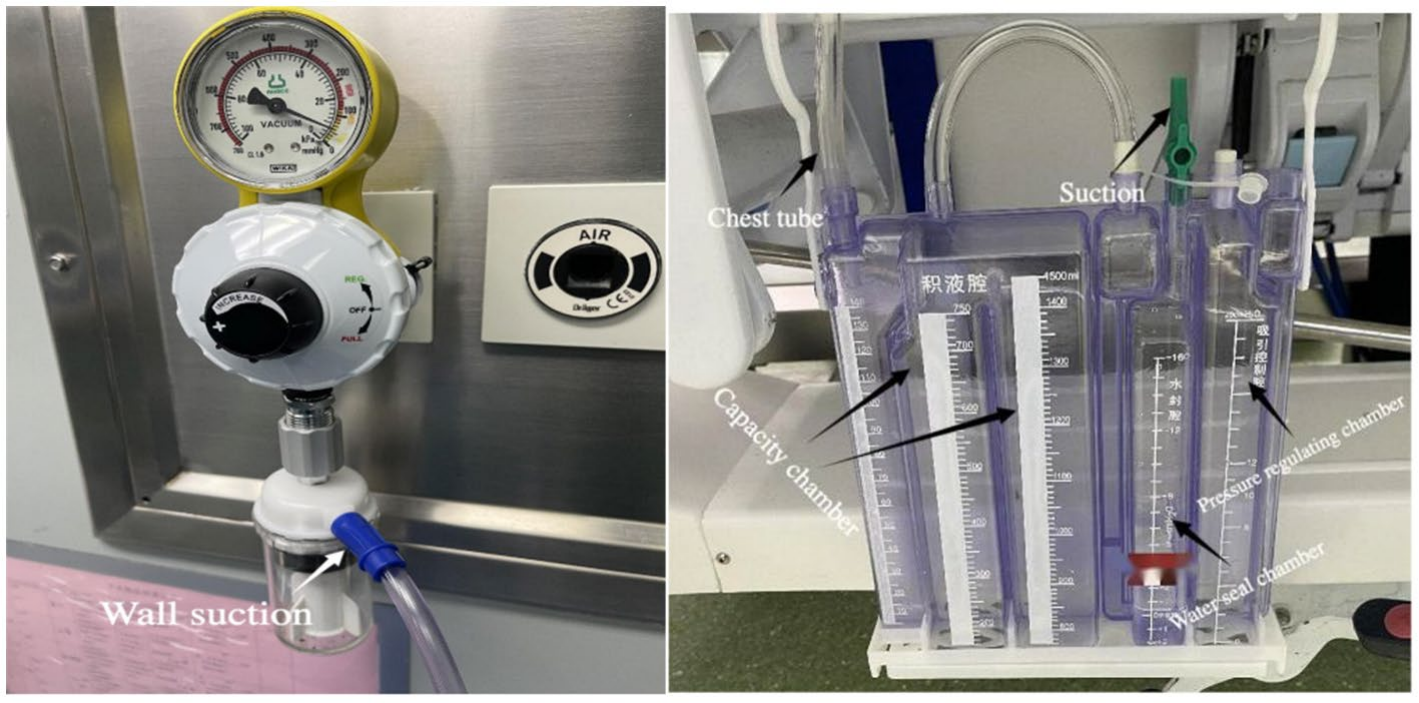
Wall suction drainage systems.

Lung cancer is one of the most common malignant tumors worldwide and is also the leading cause of cancer-related deaths (1). Video-assisted thoracoscopic surgery has been recognized as the standard treatment for early-stage lung cancer (2). Effective closed drainage (Figure 1) after lung surgery is a critical procedure for preventing complications and promoting lung re-expansion (3). With the widespread adoption of the enhanced recovery after surgery (ERAS) concept, minimizing the restrictive impact of medical interventions on patient mobility postoperatively, while ensuring drainage efficacy, has become a significant topic in perioperative thoracic surgical management (4). Traditional wall-mounted negative pressure drainage systems (Figure 2) essentially “confine” patients to the vicinity of their beds, severely impeding early ambulation, increasing risks of postoperative complications, and potentially prolonging hospital stays (5). In recent years, the invention of digital lead generation systems has solved this problem and they have the functions of objective quantification and tracking of data. However, their high cost (typically over 2,000 to 3,000 US dollars) has seriously hindered their clinical application and promotion (6). Therefore, there is an urgent need to develop an economical, practical and portable negative pressure closed thoracic drainage device.

### 1.2 Technological evolution and motivation for iteration

Our research team previously developed a first-generation integrated portable device, which innovatively combined a micro-air pump, power system, and a three-chamber drainage bottle into a compact portable unit using 3D printing technology (5). This design initially achieved the portability of the drainage apparatus. Although we discussed the advantages brought by the first generation of portable thoracic negative pressure drainage devices in the second article (7), the long-term usage cost and noise of these devices cannot be ignored either.

Consequently, we initiated the development of the second-generation device. The core objectives were to further enhance clinical applicability and user experience through a split-type design while retaining portability advantages, and to ensure an extremely simplified operational workflow that integrates seamlessly into existing clinical practices.

## 2 Materials and methods: design philosophy and implementation

### 2.1 Overview of the first-generation device (integrated design)

The core design philosophy of the first-generation device was “high integration.” Medical-grade 3D printing technology was employed to fabricate an outer casing that served both as a protective shell and a structural frame. This casing housed a precisely assembled micro negative pressure pump, a rechargeable lithium battery with its management circuit, and a dedicated compartment designed to accommodate a standard three-chamber closed drainage bottle. The bottle was fully or partially enclosed within the casing, forming an inseparable whole. In use, medical staff simply connected the chest tube as with a conventional bottle and turned on the power (5). This design fundamentally altered the form factor of the drainage setup, transforming it from a bedside fixture into a handheld, mobile, independent unit.

### 2.2 Optimizations and innovations in the second-generation device (split-type design)

**Figure 3:**
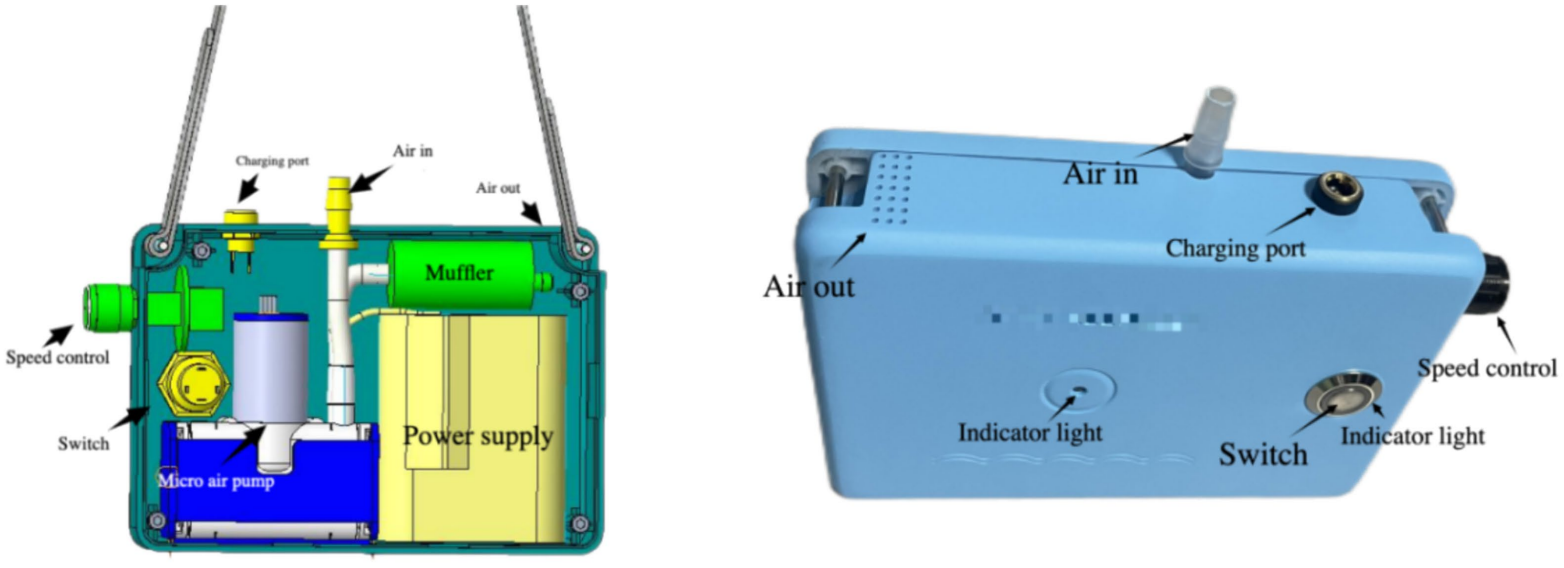
Novel portable negative pressure device.

**Figure 4:**
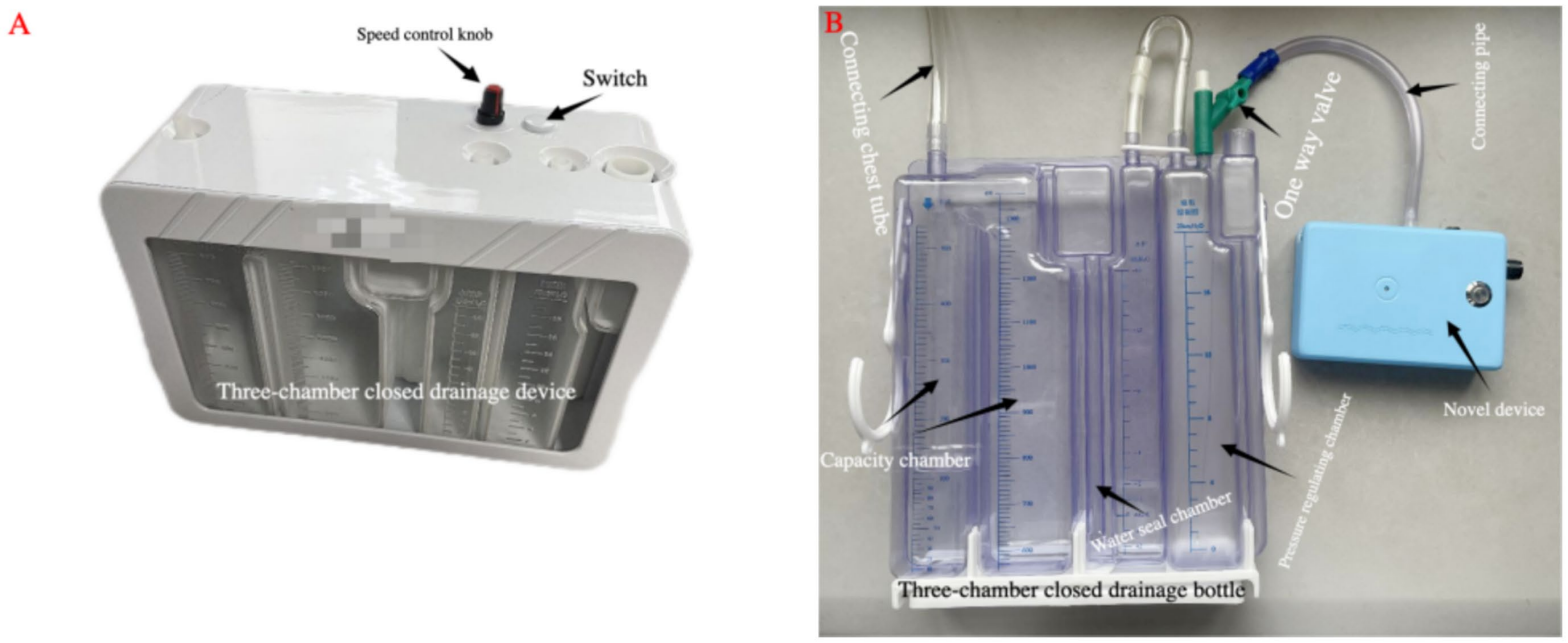
**(A)** The first-generation integrated device. **(B)** The second-generation split-type device.

The core improvement of the second-generation device is “flexible coupling.” The design philosophy shifted from “integration” to “attachment,” implemented as follows (Figures 3, 4).

#### 2.2.1 Independent host design

Similarly utilizing 3D printing, a structurally more refined and optimally laid-out portable host was manufactured. This host integrates a negative pressure system, a power supply system and a noise reduction system. The material of the pipeline interface of the main unit is polypropylene, and the material of the pipeline is silicone. The power of the rechargeable battery is 36.72 Wh.

The host no longer contains space for the drainage bottle, being designed as an independent functional module, resulting in significantly reduced volume and weight.

#### 2.2.2 Innovative connection method—clinically aligned “flexible attachment”

This represents the most innovative aspect of the second-generation device. We abandoned complex clips or mechanical locks, adopting double-sided adhesive tape and 3M hook-and-loop fasteners as the connection medium between the host and the drainage bottle.

##### 2.2.2.1 Operational workflow

Medical staff retrieves a standard three-chamber drainage bottle and connects it to the chest tube per routine. Then, as needed, the loop or hook side of the fastener is pre-applied to a suitable position on the side of the bottle, with the counterpart fixed to the back of the portable host. During use, the host is simply aligned with the bottle side and pressed on for a secure connection. It can also directly attach the side of the three-chamber closed drainage bottle and the side of the host with double-sided tape.

#### 2.2.3 Design advantages

##### 2.2.3.1 Extreme simplicity

No tools required, no new steps to learn. The sticking action is intuitive, instantly mastered by any clinician.

##### 2.2.3.2 Non-destructive compatibility

It does not alter the structure or function of the drainage bottle itself, is compatible with nearly all standard models available on the market, completely eliminating the constraint of proprietary consumables and significantly reducing usage costs.

##### 2.2.3.3 Secure and reliable

The three-chamber closed drainage bottle is a drainage device that has been tested by practice. Even if the new device malfunctions, patients can still rely on the three-chamber closed drainage bottle as a basis to ensure normal thoracic drainage.

#### 2.2.4 Simplified human-machine interface design

The core human-machine interface (HMI) philosophy is “intuitive operation, zero learning threshold.” The interface is distilled to two essential physical controls:

One-touch power/Function switch: A large, backlit button allows easy operation even with gloved or impaired hands (common postoperatively). Embedded LED indicates status: off (device off), steady green (normal operation).
The rotational speed and pressure adjustment knob: The height of the water column within the pressure regulation chamber of the three-chamber closed drainage bottle indicates the negative pressure intensity, with clearly marked pressure gradations (e.g., −5, −10, −15, −20 cm H₂O). The rotational speed knob regulates the airflow rate, and operation is straightforward, requiring only rotation of the knob.

This design compresses the learning curve to under 1 min, empowering patients and caregivers for safe management in non-professional settings like home care, supporting ERAS principles of patient empowerment and continuity of care.

#### 2.2.5 Concurrent performance and cost optimization

Noise can affect patients’ recovery (8). In the split architecture, we have carried out coordinated optimization of the pump, airway and circuit, taking noise reduction as a key performance indicator. The final noise control data is as follows. However, due to the formation of water bubbles when the three-chamber closed drainage bottle is under negative pressure suction, the noise data may be disturbed.

The modular design facilitates assembly and maintenance. Combined with the selection of general-purpose commercial off-the-shelf (COTS) components and scale production forecasts, the target unit cost for the host was set around 500 Chinese Yuan (about 70 USD). Consumables are limited to generic three-chamber bottles (about 45 Chinese Yuan, about 6.3 USD each) and inexpensive adhesive/fastener strips, resulting in extremely low per-use costs.

### 2.3 Performance testing protocol

The second-generation prototype underwent systematic laboratory testing, including:

Physical parameters: Dimensions, weight.
Core performance: Maximum negative pressure, host unit maximum flow, noise test at 3 L/min, battery life.
Connection reliability: The floating time of the new equipment under the double-sided tape connection method has exceeded 72 h.
Clinical simulation: Conduct a complete operation process drill *in vitro* to evaluate the integration with the existing clinical workflow.

## 3 Results

### 3.1 Final device form and technical specifications

The second-generation novel portable split-type negative pressure drainage device consists of two parts: (1) an independent functional host, and (2) a standard three-chamber closed drainage bottle. They are rapidly joined or separated via the intermediary (hook-and-loop/adhesive).

Key performance parameters from testing are as follows (Table 1):

Host dimensions: 95.6 mm × 35 mm × 139.6 mm.
Host weight: about 400 g.
Maximum negative pressure: close to −20 cm H₂O.
Host unit noise: <55 dB @ 3 L/min flow.
Host unit maximum flow: about 9 L/min.
Battery life: about 6–10 h (depending on set pressure and air leak).
Connection method: 3M hook-and-loop/Double-sided tape, the double-sided tape suspension test indicates that the suspension time of the new device has exceeded 72 h.
Cost estimate: Host ~about 500 Chinese Yuan/unit (about 70 USD); Single-use consumables (drainage bottle) about 45 Chinese Yuan (about 6.3 USD).

**Table 1:** Key performance parameters.

Host dimensions (mm)	95.6 × 35 × 139.6
Host weight (g)	About 400
Maximum negative pressure [height of H₂O (cm)]	Close to 20
Host unit noise (dB)	<55 @ 3 L/min flow
Host unit maximum flow (L/min)	About 9
Battery life (hours)	About 6–10
Connection method	3M hook-and-loop/Double-sided tape
Cost estimate (USD)	Host unit (about 70)/Single-use consumables (drainage bottle about 6.3)

### 3.2 Validation of clinical operability and user-friendliness

Simulated clinical operation assessments demonstrated:

Minimal learning curve: The intuitive one-touch and rotary controls enabled medical staff, patients, and family caregivers to master all operations within 1 min. The “stick, press, turn” logic was immediately understood without complex manuals.
Seamless workflow integration and extended application: The preparatory phase remained the routine bottle priming and tube connection. The only added steps were “attaching the host” and “pressing the power button,” taking less than 15 s. This simplicity is particularly conducive to patient self-management and family-assisted care postoperatively, significantly enhancing the device’s applicability and safety in non-critical care environments (e.g., general wards, rehab facilities, home).

### 3.3 Cost–benefit analysis

Compared to the first-generation integrated design, the second-generation split-type design eliminates the need for a custom complex structure to house the bottle per device. This simplifies the 3D-printed host structure, reducing mold costs and production complexity. More importantly, it completely removes reliance on “proprietary drainage bottles,” aligning consumable costs directly with the market, establishing an economic foundation for widespread adoption.

## 4 Discussion

The fundamental concept of closed thoracic drainage is to effectively remove accumulated air or fluid from the thoracic cavity through a drainage system, thereby maintaining negative pressure in the thoracic cavity and promoting lung re-expansion. Over the past several hundred years, this technique has undergone continuous improvement and refinement, experiencing significant development stages from single-chamber systems, two-chamber systems, three-chamber systems to digital drainage systems. Each generation of the system has unique structural and functional designs, as well as certain advantages and limitations (9). The features comparison of the novel device and the digital device (mainly Thopaz) are shown in Table 2. The negative pressure intensity of the three-chamber closed drainage bottle main depends on the height of the water column in the pressure regulating chamber. The one-way valve in the pressure regulating chamber controls the speed of air flow. The negative pressure source only serves as a suction function. The essential feature of our new device is that it has transformed the wall-mounted negative pressure source into a portable one. The maximum height of the water column in the pressure regulating chamber of the commonly used three-chamber closed drainage bottle is approximately 20 cm, so the maximum negative pressure intensity of ours is close to 20 cm of water column. However, as the suction proceeds, the height of the water column in the pressure regulating chamber will decrease due to water evaporation, and the nurse needs to replenish water to maintain the negative pressure intensity. Although the novel device and the pleural fluid will increase the overall weight of the device, fortunately, the novel device is not heavy, and it is estimated that it will have little impact on the patient’s activities. During the perioperative management of thoracic tubes in lung surgeries, the thoracic drainage device plays an indispensable and crucial role. A large number of studies have confirmed that continuous and stable negative pressure drainage can effectively reduce postoperative complications and significantly improve the postoperative recovery quality of patients (10–12). In fact, in our latest article, we have already completed the clinical concept validation of the mechanism of the modular closed thoracic drainage device (13). In this study, we further developed it into a portable host unit and explored it.

**Table 2:** Features of the novel device and digital device (mainly Thopaz).

Features	Portability	Pressure regulation mechanism	Digital monitoring	Objective quantification
Digital device	Yes	Mechanical pressure regulation (dry type)	Yes	Yes
Novel device	Yes	Water column pressure regulation (wet type)	No	No

### 4.1 Design philosophy: clinical-centric unobtrusive innovation

The success of this optimization lies in its “unobtrusive innovation” philosophy. The second-generation device introduces no complex interfaces, locks, or setup procedures that would alter clinician habits (4). By leveraging the most straightforward and reliable attachment method and an extremely intuitive physical HMI (one-touch switch and rotary knob), it “adds” innovative functionality onto an existing mature product (the standard bottle), minimizing operational complexity. This approach greatly reduces barriers to clinical adoption and enhances acceptance across the spectrum from professionals to patient families, which is crucial for medical devices achieving seamless transition from hospital to home.

### 4.2 Advantages of the split-type design and connection method

The split-type design employing hook-and-loop/adhesive offers multiple advantages:

Maintenance and cleaning: The host can be detached separately for charging, maintenance, or use with other patients (after changing adhesive), while the bottle is disposed of as medical waste, complying with infection control.
Upgradability and iteration: Future technological upgrades to the host (e.g., adding digital display, wireless transmission) require only replacing the host unit, preserving the investment in the bottle system.
Patient psychological acceptance: The transparent standard bottle allows patients and staff to visually observe effluent characteristics and air leaks, preserving the reassurance of the traditional method and avoiding potential apprehension associated with fully enclosed devices.

### 4.3 Alignment of performance parameters with clinical value

The achieved performance parameters match core clinical needs: providing sufficient negative pressure to facilitate lung re-expansion and manage air leakage, while creating a relatively quiet environment when the patient moves. The weight and battery life effectively support early mobilization protocols within the ERAS framework (14).

### 4.4 Limitations and future perspectives

This study is currently at the prototype development and laboratory testing stage, the data of the prototype may have some deviations. However, this will not affect our research conclusion. The primary limitation is the lack of large-sample clinical data on effectiveness, safety, and health economics. Future research directions include: (1) Conducting prospective clinical studies to compare its efficacy, complication rates, patient satisfaction, and length of stay against traditional systems; (2) Further optimizing the host’s industrial design for improved water/dust resistance; (3) Exploring integration of micro-sensors for automated effluent volume estimation and alarm triggers.

## 5 Conclusion

**Figure 5:**
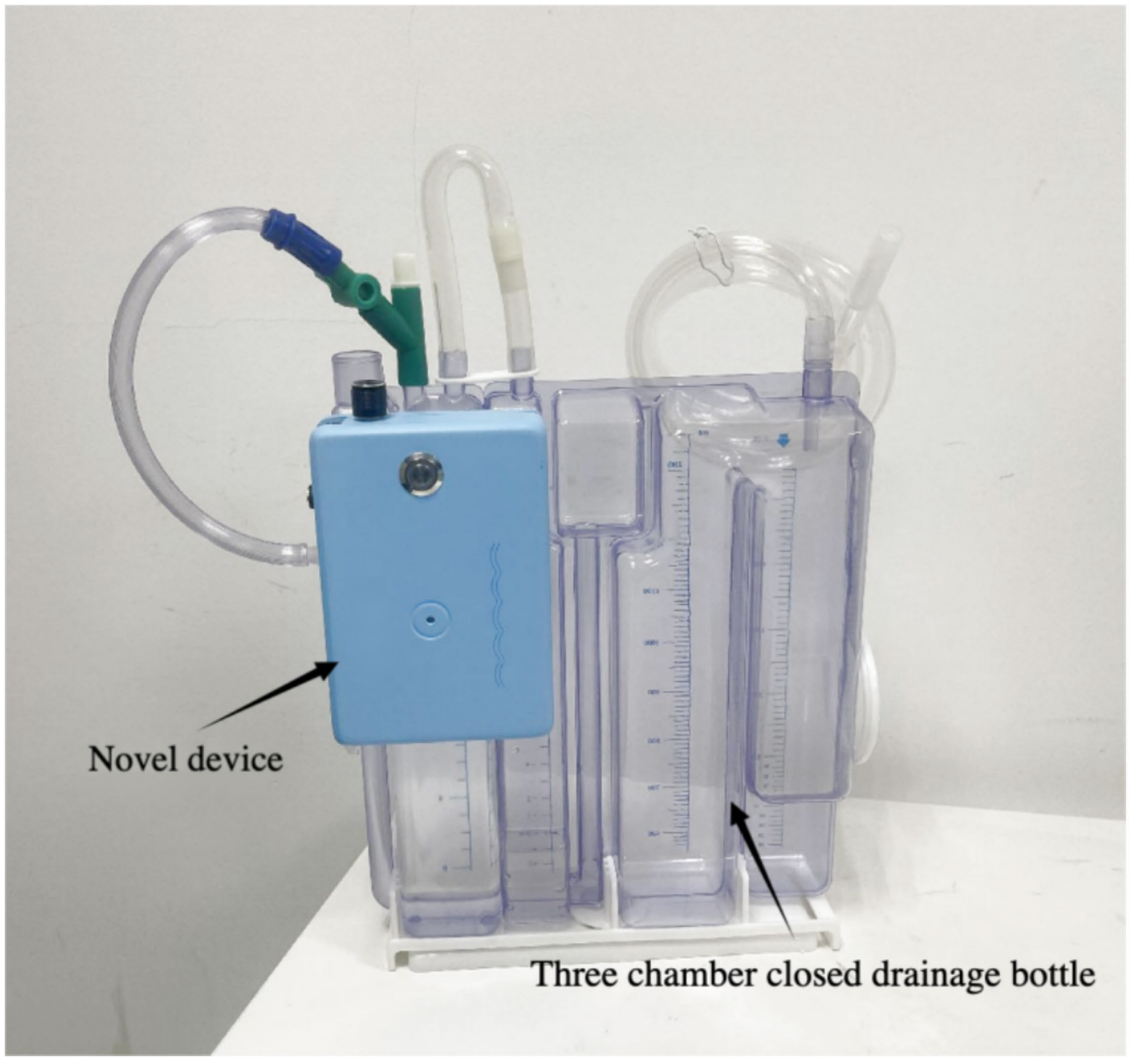
Novel portable split-type negative pressure thoracic drainage device.

This study proposed the concept of a novel type of portable negative pressure thoracic drainage device and designed and manufactured a sample. In the next step, we will continue to improve this novel device and plan to apply it in clinical practice (see Figure 5).

## Data Availability

The original contributions presented in the study are included in the article/supplementary material, further inquiries can be directed to the corresponding authors.
